# Mortality in sepsis versus non-sepsis induced acute lung injury

**DOI:** 10.1186/cc8048

**Published:** 2009-09-16

**Authors:** Jonathan E Sevransky, Gregory S Martin, Carl Shanholtz, Pedro A Mendez-Tellez, Peter Pronovost, Roy Brower, Dale M Needham

**Affiliations:** 1 Division of Pulmonary and Critical Care, Johns Hopkins University, 5501 Hopkins Bayview Circle Baltimore, MD 21224 USA; 2 Division of Pulmonary and Critical Care Emory University 615 Michael Street, Atlanta Georgia, 30322, USA; 3 Division of Pulmonary and Critical Care, University of Maryland, 10 South Pine Street Baltimore MD, 21201, USA; 4 Department of Anesthesiology and Critical Care, Johns Hopkins University, 600 North Wolfe Street Baltimore, MD, 21287, USA

## Abstract

**Introduction:**

Sepsis-induced acute lung injury (ALI) has been reported to have a higher case fatality rate than other causes of ALI. However, differences in the severity of illness in septic vs. non-septic ALI patients might explain this finding.

**Methods:**

520 patients enrolled in the Improving Care of ALI Patients Study (ICAP) were prospectively characterized as having sepsis or non sepsis-induced ALI. Biologically plausible risk factors for in-hospital death were considered in multiple logistic regression models to evaluate the independent association of sepsis vs. non-sepsis ALI risk factors with mortality.

**Results:**

Patients with sepsis-induced ALI had greater illness severity and organ dysfunction (APACHE II and SOFA scores) at ALI diagnosis and higher crude in-hospital mortality rates compared with non-sepsis ALI patients. Patients with sepsis-induced ALI received similar tidal volumes, but higher levels of positive end expiratory pressure, and had a more positive net fluid balance in the first week after ALI diagnosis. In multivariable analysis, the following variables (odds ratio, 95% confidence interval) were significantly associated with hospital mortality: age (1.04, 1.02 to 1.05), admission to a medical intensive care unit (ICU) (2.76, 1.42 to 5.36), ICU length of stay prior to ALI diagnosis (1.15, 1.03 to 1.29), APACHE II (1.05, 1.02 to 1.08), SOFA at ALI diagnosis (1.17, 1.09 to 1.25), Lung Injury Score (2.33, 1.74 to 3.12) and net fluid balance in liters in the first week after ALI diagnosis (1.06, 1.03 to 1.09). Sepsis did not have a significant, independent association with mortality (1.02, 0.59 to 1.76).

**Conclusions:**

Greater severity of illness contributes to the higher case fatality rate observed in sepsis-induced ALI. Sepsis was not independently associated with mortality in our study.

## Introduction

Acute lung injury (ALI) and sepsis have a close relation in the intensive care unit (ICU) setting. Sepsis is the most frequent risk factor for the development of ALI [1]. Moreover, up to 50% of patients admitted to an ICU with sepsis develop ALI [2].

Patients with sepsis-induced ALI have a higher case fatality rate than patients with other risk factors for ALI [1,3]. However, it is unclear if the higher case fatality rate is related to patient's co-morbidities, severity of illness, or the etiology of ALI. For example, patients with trauma versus sepsis as their risk factor for ALI tend to have lower case fatality rates. However, patients with trauma-related ALI also tend to be younger, with fewer co-morbid conditions and lower severity of illness compared with patients with sepsis-induced ALI [4,5].

A recent study has suggested that that sepsis is not independently associated with mortality from ALI [6]. Our objective is to evaluate whether a risk factor of sepsis is independently associated with mortality in a large cohort of racially diverse ALI patients. A secondary objective is to evaluate clinical and treatment characteristics in this cohort. We have previously demonstrated that in patients with sepsis-induced ALI, a pulmonary versus nonpulmonary source of infection is not independently associated with patient mortality. [7]. Hence, we considered all sources of sepsis-induced ALI together in this evaluation. The purpose of this study is to examine whether the presence of sepsis as a risk factor for ALI is independently associated with mortality in a large representative multi-site cohort of ALI patients.

## Materials and methods

### Study population

This is a retrospective analysis of consecutive ALI patients enrolled into a multi-site prospective cohort study during a three-year period ending in October 2007 [8]. In this study, 12 ICUs at 4 teaching hospitals enrolled consecutive mechanically ventilated patients who met the American-European consensus criteria for ALI [9]. Relevant exclusion criteria included: pre-existing illness with a life expectancy of less than six months; transfer to a study site ICU with pre-existing ALI of more than 24 hours' duration; more than five days of mechanical ventilation prior to ALI diagnosis; and limitations in ICU care (e.g. no vasopressors) at eligibility.

### Primary outcome and exposure variables

The primary study outcome was in-hospital mortality. The primary exposure variable was sepsis versus non sepsis as the etiology of ALI with this classification prospectively obtained based on documentation in the medical record for the ICU physicians. Patients with pulmonary or non-pulmonary infections were classified as having sepsis. Any uncertainty in the classification of the primary exposure variable was addressed by an ICU investigator at each study site based on review of the medical record and discussion with the treating ICU physicians.

### Patient demographic and severity of illness variables

Patient-related exposures of interest (independent variables) included patient demographics and several measures of severity of illness. These included: Acute Physiology and Chronic Health Evaluation (APACHE) II at ICU admission [10]; lung injury severity at onset of ALI (lung injury score (LIS) calculated based on the number of affected quadrants on chest x-ray, positive end expiratory pressure (PEEP) and partial pressure of arterial oxygen/fraction of inspired oxygen ratio [11-13]); and the organ failure score at onset of ALI (Sequential Organ Failure Assessment (SOFA) score) [14]. Length of stay in hospital and ICU prior to ALI diagnosis was also included as an independent variable.

Race was determined by chart review and examination of the patient. We limited our analysis of race to white and black because of the low number of enrolled patients of other races. (12 of 520, including 7 Asian, 3 other and 2 unknown)

### ICU management exposure variables

Data were collected on the following variables related to the ICU management of ALI patients: tidal volume at day 1 after ALI diagnosis; PEEP at day 1 after ALI diagnosis; and net fluid balance during the first seven days after ALI diagnosis [11,12]. Tidal volume and PEEP were abstracted from medical records using settings/measurements for 6:00 AM on the day after ALI diagnosis with tidal volume reported in ml/kg of predicted body weight as per the acute respiratory distress syndrome network calculations [11,15]. If tidal volume was not available at that time point, data was imputed from the earliest timepoint 12 or 24 hours before; most patients who did not have tidal volumes had been switched to a mode of ventilation (high frequency oscillatory ventilation) for which there was no PEEP available. (Imputation required for 40 patients with no data available for 6 patients; tidal volume and PEEP were generally not available because patients had been switched to high frequency oscillatory ventilation for which these ventilator settings are not available). Cumulative fluid balance was calculated during the first seven days that patients were alive and in the ICU based on the total intravenous and oral intake less the total urinary, gastrointestinal, dialysis and other fluid losses as applicable.

### Statistical analysis

Continuous variables were reported as medians, categorical variables as proportions, and compared using Wilcoxin's rank sum, t-tests, and chi-squared tests, as appropriate. Biologically plausible risk factors for in-hospital death were considered in multiple logistic regression models if *P *< 0.1 in a univariable analysis. In the final multivariable model, we confirmed goodness of fit (using Pearsons chi-square and Hosmer-Lemeshow tests) and absence of colinearity (evaluated using variance inflation factors) between all demographic, severity of illness and ICU management exposure variables. We confirmed that there were no important statistical interactions of sepsis versus non-sepsis with clinically relevant exposure variables selected on an *a priori *basis by including individual multiplicative terms in the multivariable logistic regression models. All analyses were performed using Stata 10.0 software (Stata Corporation, College Station, TX, USA). A two-sided *P *< 0.05 was used to determine statistical significance.

### Informed consent

A two-step process incorporating delirium screening was used to obtain informed consent from patients. Patients were screened daily for the presence of delirium using the validated screening tools Richmond Agitation-Sedation Scale (RASS) and Confusion Assessment Method for the Intensive Care Unit (CAM-ICU). The Institutional Review Board granted a waiver of consent for collection of observational data on eligible patients. Patients were approached for consent when RASS and CAM-ICU data demonstrated resolution of delirium, and after assessment and determination of competency. The institutional review boards of Johns Hopkins University and all participating sites approved this study

## Results

Of the 520 ALI patients enrolled in the study, 383 (74%) had sepsis as the primary risk factor for ALI, with 137 (24%) having other causes including 64 (12%%) with aspiration, 18 (3%) with pancreatitis, 8 (3%) with multiple transfusion, 12 (2%) with trauma, 15 (3%) with unknown causes and 7 (1%) with other causes. Patients with sepsis-induced ALI had greater severity of illness and organ dysfunction (APACHE II and SOFA scores) and higher crude in-hospital mortality rates (50 versus 33%) compared with non sepsis-induced ALI patients (Table 1). There were no significant differences in patients in age, gender or lung injury score at ALI diagnosis in patients with sepsis versus no-sepsis ALI risk factors.

**Table 1:** Patient demographics, clinical characteristics, and in-hospital mortality

	Sepsis n = 383*	Non-sepsis n = 137*	*P *value**
Age, years	53 (43, 63)	50 (40, 64)	0.12
Female gender	47%	46%	0.78
Race/Ethnicity			
Black	43%	27%	0.01
White	55%	70%	
Other	2%	3%	
Medical ICU	86%	63%	<0.0001
ICU LOS prior to ALI diagnosis	1 (0, 2)	1 (0, 2)	0.84
Hospital LOS prior to ALI diagnosis	2 (1, 6)	2 (1, 5)	0.28
APACHE II score at ICU admission	28 (21, 34)	22 (17, 28)	<0.0001
SOFA at ALI diagnosis	10 (6, 13)	8 (7, 11)	<0.0001
Lung Injury Score at ALI diagnosis	2.0(1.7, 3.0)	2.0 (1.7, 2.7)	0.64

* Continuous variables are presented as median with interquartile range and categorical variables as proportions.

**Calculated using student's t-test for continuous data that appeared normally distributed, Wilcoxin rank sum for variables that did not appear normally distributed, and the chi-squared test for categorical data.

ALI = acute lung injury; APACHE = Acute Physiology and Chronic Health Evaluation; ICU = intensive care unit; LOS = length of stay; SOFA = Sequential Organ Failure Assessment.

Of the total cohort, 38% were black, 59% white and 3% other. Black patients were more likely than white patients to have sepsis (43% versus 27%) as a risk factor for ALI (*P *= 0.01). Demographic characteristics of white and black ALI patients can be seen in Table 2.

**Table 2:** Patient demographics, clinical characteristics for white and black ALI patients

	White n = 308*	Black n = 200*	*P *value**
Age, years	53 (43, 63)	50 (40, 64)	0.12
Female gender	47%	46%	0.78
Sepsis	68%	81%	0.002
Medical ICU	74%	89%	<0.001
Hospital LOS prior to ALI diagnosis	3 (1, 6)	2 (1,6)	0.05
ICU LOS prior to ALI diagnosis	1 (0, 2)	0 (0, 1)	0.009
APACHE II	26 (20,33)	26 (20,34)	0.7
SOFA at ALI diagnosis	9 (7, 12)	9.5 (6, 12)	0.97
Lung Injury Score	2(1.7, 2.7)	2(1.7, 3)	0.23
Mortality in-hospital	45.5%	45.1	.93

* Continuous variables are presented as median with interquartile range and categorical variables as proportions. Does not include the 12 patients with different racial backgrounds

**Calculated using Student's t-test for continuous data that appeared normally distributed, Wilcoxin rank sum for variables that did not appear normally distributed, and the chi-squared test for categorical data.

ALI = acute lung injury; APACHE = Acute Physiology and Chronic Health Evaluation; ICU = intensive care unit; LOS = length of stay; SOFA = Sequential Organ Failure Assessment.

Patients with sepsis-induced ALI were treated in the ICU with higher PEEP on day 1 and had a greater net fluid balance in the first week after ALI diagnosis compared with non-sepsis-induced ALI (Table 3). This greater net fluid balance in the sepsis-induced ALI patients was present on days 1 to 3, but not days 4 to 7 (data not shown). Tidal volumes per kilogram of predicted body weight were similar between groups.

**Table 3:** Ventilation and fluid therapy in ICU

Parameter	Sepsis*	Non-Sepsis*	P value**
Tidal volume at day 1, mL/kg PBW***	6.7 (5.8, 7.9)	6.4 (4.9, 7.8)	0.16
PEEP on day 1, cmH_2_0***	8 (5, 10)	5 (5, 10)	0.0004
PaO_2 _on day1	81 (66-107)	87 (66-125)	0.15
Cumulative fluid balance during first 7 days after ALI onset, Liters	9.8 (3.9, 17)	7.1 (1.9, 13)	0.004

* Continuous variables are presented as median with interquartile range.

**Calculated using Student's t-test for continuous data that appeared normally distributed, and the Wilcoxin rank sum for variables that did not appear normally distributed.

***For the sepsis and non-sepsis groups, data was missing, and could not be imputed, for tidal volume and PEEP for six patients, five in the sepsis group and one in the non-sepsis group.

PBW = predicted body weight; PEEP = positive end-expiratory pressure.

In univariable analysis, most of the variables with a clinically plausible association with mortality were significantly associated with mortality (Table 4). Sepsis as a risk factor for ALI was associated with mortality in univariable analysis (odds ratio, 95% confidence interval) (2.06, 1.37 to 3.09). In multivariable analysis, several variables (odds ratio, 95% confidence interval) had independent association with mortality: age (1.04, 1.02 to 1.05), admission to a medical ICU (2.76, 1.42 to 5.36) ICU length of stay prior to ALI diagnosis (1.15, 1.03 to 1.29), APACHE II at ICU admission (1.05, 1.02 to 1.08), SOFA (1.17, 1.09 to 1.25), LIS (2.33, 1.74 to 3.12)and fluid balance in the first week after ALI diagnosis (1.06, 1.03 to 1.09) were independently associated with mortality (Table 4). In this multivariable model, sepsis was not independently associated with mortality (1.02, 0.59 to 1.76).

**Table 4:** Exposures associated with in-hospital mortality in 520 patients with ALI

	Univariable*		Multivariable*	
**Exposure**	**Odds ratio** **(95% CI)**	***P *value**	**Odds ratio** **(95% CI)**	***P *value**

Age, years	1.03 (1.02-1.04)	<0.0001	1.04 (1.02-1.05)	<0.001
Ethnicity	0.98 (0.69-1.4)	0.93		
Medical ICU	2.08 (1.32-3.30)	0.002	2.76(1.42-5.36)	0.003
ICU LOS prior to ALI diagnosis	1.09 (1.01-1.19)	0.025	1.15 (1.03-1.29)	0.014
Hospital LOS prior to ALI diagnosis	1.03 (1.01-1.06)	0.008	1.03 (0.99-1.05)	0.06
APACHE II at ICU admission	1.08 (1.06-1.11)	<0.0001	1.05 (1.02-1.08)	0.003
Lung Injury Score at ALI diagnosis	2.23 (1.77-2.81)	<0.0001	2.33 (1.74-3.12)	<0.001
SOFA at ALI diagnosis	1.26 (1.19-1.33)	<0.0001	1.17 (1.09-1.25)	<0.001
PEEP at day 1 after ALI, cmH_2_0	1.14 (1.09-1.19)	<0.0001	1.04 (0.98-1.10)	0.15
Cumulative fluid balance in first 7 days after ALI diagnosis, Liters	1.06 (1.04-1.09)	<0.0001	1.06 (1.03-1.09)	<0.001
Sepsis vs. non-sepsis ALI risk factor	2.06 (1.37-3.09)	0.001	1.02 (0.59-1.76)	0.61

* Calculated using logistic regression analysis. The odds ratio indicates the increased odds of in-hospital mortality for a one unit increase in each continuous exposure variable or for sepsis vs. non-sepsis for this binary exposure variable.

ALI = acute lung injury; APACHE = Acute Physiology and Chronic Health Evaluation; CI = confidence interval; ICU = intensive care unit; LOS = length of stay; PEEP = positive end-expiratory pressure; SOFA = Sequential Organ Failure Assessment.

## Discussion

In our multi-site study of 520 ALI patients, those with sepsis vs. non-sepsis-induced ALI had a significantly higher crude mortality rate. However, after adjustment for patient demographics, severity of illness and clinical factors, sepsis as a risk factor for ALI was not independently associated with mortality. These results suggest that the higher case fatality rate in patients with sepsis-induced ALI may be explained primarily by a greater severity of illness.

There are few studies that examine the attributable risk of sepsis as a predisposing factor for ALI. Cooke and colleagues examined a cohort of 1113 ALI patients admitted to hospitals in King County, Washington, USA [6]. Although sepsis as an ALI risk factor was predictive of mortality in univariable analysis, it was not predictive of mortality in their multivariable model. Of note, less than 10% of the patients in their cohort were black [6] Black patients are more likely to develop sepsis, and have a higher case fatality rate from ALI [16,17]. Our study in a racially diverse cohort of white and black patients also found that sepsis as an ALI risk factor was not predictive of mortality. In addition, Estenssoro and colleagues examined risk factors for mortality in 217 Hispanic ALI patients [18]. Although sepsis also was not independently associated with mortality, they included patients who developed sepsis after admission and thus were not specifically evaluating the association of sepsis as an ALI risk factor on in-hospital mortality [18].

Our results are also consistent with the results of Sakr and colleagues, who demonstrated that sepsis was predictive of mortality in univariate but not multivariate analysis in European ICUs [19]. Of note, more than one-third of ALI patients in that cohort had mean tidal volumes greater than 8 cc/kg [19]. In their model, both fluid balance over the first four days after ALI diagnosis and a composite exposure based on tidal volume, plateau pressure and PEEP were independently predictive of outcome. Consistent with their findings and those of Payen and colleagues [20], we also found that net fluid balance over the first week after ALI diagnosis was predictive of mortality.

Our study has several potential limitations. First, as an observational study, inferences from our findings are dependent on complete adjustment for all relevant confounders. As patients cannot be randomized to their risk factor for ALI, an observational study is the only way in which we can evaluate the potential independent mortality effects of sepsis-induced versus non-sepsis-induced ALI in humans. In this prospective study, we adjusted for plausible patient and treatment-related risk factors for mortality, specifically adjusting for differences in severity of illness using three different ICU measures which were not colinear, and all remained statistically associated with mortality in our final multivariable model. Second, we enrolled patients from teaching hospitals in one geographic area, and thus the results may not be generalizable to other hospitals in other regions. However, our results appear to be consistent with published studies from other regions, including academic and private hospitals as well as teaching hospitals in Argentina [6,18]. Third, while the mortality rates for our observational trial for both sepsis and non-sepsis-induced ALI are higher than in some interventional trials, this higher mortality rate has been seen in other observational trials [21].

We cannot exclude the possibility of misclassification bias in the diagnoses of ALI and sepsis. However, our participating study sites have significant experience with these critical illnesses and have participated in many previous clinical trials enrolling patients with both sepsis and ALI. It is possible that misclassification bias remains. In such a case, this bias might be non-differential, potentially obscuring a true difference in mortality between the sepsis and non-sepsis groups. Finally, if therapies that improve patient mortality rates were delivered at a higher rate (intentionally or unintentionally) to patients with sepsis-induced or non-sepsis-induced ALI, we could miss a potential true difference in between groups for our mortality outcome. Of note, patients with sepsis-induced versus non-sepsis-induced ALI had a greater net fluid balance over the first week in the ICU, which is related to the initial resuscitation of patients with sepsis. However, while a fluid conservative strategy has been associated with increased days alive and off the ventilator, it has not been shown to influence ALI mortality rates [12].

## Conclusions

Sepsis-induced ALI is not independently associated with mortality after adjustment for the greater severity of illness in these patients versus those with a non-sepsis risk factor for lung injury. In conjunction with the results from other studies, our research suggests that severity of illness, rather than the precipitating risk factor for ALI, should be considered in making treatment decisions and predicting outcome for these patients.

## Key messages

• Patients with sepsis-induced ALI had greater severity of illness and higher crude in-hospital mortality rates compared with non-sepsis-induced ALI patients.

• In multivariable analysis, severity of illness measures, admission to a medical ICU and length of ICU stay prior to developing ALI were all associated with in-hospital mortality. Sepsis as a risk factor for ALI was not independently associated with mortality in a racially diverse cohort of 520 patients.

• More black patients had sepsis as a risk factor for ALI, and were more likely to be admitted to a medical ICU. Black patients had similar severity of illness scores, and crude inpatient mortality rates. Race was not independently associated with mortality rates.

## Abbreviations

ALI: acute lung injury; APACHE: Acute Physiology and Chronic Health Evaluation Score; CAM-ICU: Confusion Assessment Method for the Intensive Care Unit; ICU: intensive care unit; LIS: lung injury score; PEEP: positive end-expiratory pressure; RASS: Richmond Agitation-Sedation Scale; SOFA: Sequential Organ Failure Assessment.

## Competing interests

The authors declare that they have no competing interests.

## Authors' contributions

All authors made substantial contribution to the study design and methods. JES and DMN planned the study. JES performed the data analysis. JES drafted the manuscript and all other authors critically revised it for important intellectual content. All authors approved the final version of the manuscript for publication.
